# A real-time PCR method for quantification of the total and major variant strains of the deformed wing virus

**DOI:** 10.1371/journal.pone.0190017

**Published:** 2017-12-19

**Authors:** Emma L. Bradford, Craig R. Christie, Ewan M. Campbell, Alan S. Bowman

**Affiliations:** Institute of Biological and Environmental Sciences, School of Biological Sciences, University of Aberdeen, Aberdeen, United Kingdom; University of North Carolina at Greensboro, UNITED STATES

## Abstract

European honey bees (*Apis mellifera*) are critically important to global food production by virtue of their pollination services but are severely threatened by deformed wing virus (DWV) especially in the presence of the external parasite *Varroa destructor*. DWV exists as many viral strains with the two major variants (DWV-A and DWV-B) varying in virulence. A single plasmid standard was constructed containing three sections for the specific determination of DWV-A (VP2 capsid region), DWV-B (IRES) and a conserved region suitable for total DWV (helicase region). The assays were confirmed as specific and discriminatory with limits of detections of 25, 25 and 50 genome equivalents for DWV-A, DWV-B and total-DWV, respectively. The methods were successfully tested on *Apis mellifera* and *V*. *destructor* samples with varying DWV profiles. The new method determined a more accurate total DWV titre in samples with substantial DWV-B than the method currently described in the COLOSS Beebook. The proposed assays could be utilized for the screening of large quantities of bee material for both a total DWV load overview along with more detailed investigations into DWV-A and DWV-B profiles.

## Introduction

The European Honeybee (*Apis mellifera*) is the most economically important global pollinator, estimated to pollinate crops worth $10billion in the United States of America every year, while in the European Union insect pollination is worth around €14.6billion annually [1,2]. In 2005, insect pollination represented 9.5% of the total global value of agricultural production, which equated to €153billion [3]. Colony numbers across Central Europe and the USA have been declining since the mid-1980s [4,5]. One of the main factors that impacts colony loss are viral infections and their vector, *Varroa destructor*. *V*. *destructor* is an ectoparasite of *A*. *mellifera* which jumped host from the Asian Honeybee (*Apis cerana*) to *A*. *mellifera* in the 1960s and subsequently rapidly spread globally [6–8]. At an individual level, *V*. *destructor* infestation has been shown to cause immunosuppression and weight loss due to the mites haemolymph feeding activity [9,10]. At a colony level workers, from infested colonies have reduced vitellogenin levels, which is used as a protein store for overwintering bees resulting in lower survival to spring [11]. High individual DWV titres have been found to be associated with overwinter colony losses [12,13].

Twenty four viruses have been identified to date in *A*. *mellifera*, eight of which have been found to be associated with *V*. *destructor*-vectored transmission, including deformed wing virus (DWV) [14,15]. DWV is a member of the picorna-like insect virus family *Iflaviridae*, consisting of a 30nm icosahedral particle with a single, positive strand RNA genome [15–17]. Prior to the arrival of *V*. *destructor* DWV existed as an endemic covert infection with no visible symptoms in *A*. *mellifera* [15,16,18,19]. However, in the presence of *V*. *destructor*, overt DWV infections occur giving rise to *A*. *mellifera* with deformed wings, bloated, shortened and discoloration of the abdomen and shortened life spans [15]. DWV has been demonstrated to replicate within whole *V*. *destructor* [20,21] and isolated tissues [22] rather than acting simply as a mechanical vector. Several studies have highlighted that after *V*. *destructor*’s arrival in a new location the DWV viral diversity reduces and viral loads increase [23,24]. Like many RNA viruses, DWV exists as many related variant strains [16,23,25]. Historically, the main variants discussed when considering DWV infection are: the classical DWV strain (DWV-A) and the *Varroa destructor* virus-1 (VDV-1) strain (DWV-B) and recombinants between the these two variants [16,23,25,26]. Recent molecular analysis of *A*. *mellifera* samples from across the UK, has shown DWV-B to be widespread [26]. Detailed global distribution of DWV-B is currently unknown, with a study in the USA not detecting significant amounts of DWV-B [27].

The main method currently utilized in viral load molecular analysis is quantitative PCR (qPCR). Viral loads can be compared by using the Cq (quantification cycle) value; with low Cq values resulting from large amounts of the amplified product indicating higher viral loads [28]. Viral loads of four *A*. *mellifera* viruses have been calculated in qPCR assays by the use of standard curves produced from cDNA amplicons following spectrophotometric quantifications [29,30]; however this method requires positive samples to naturally occur during sample collection. To increase the reproducibility and the absolute quantification in qPCR assays plasmid standards containing target sequences can be employed. External plasmid standards are routinely used for the absolute quantification of genes and infection status [31–34]. Currently, there are individual external plasmid standards designed for three honeybee viruses: Chronic bee paralysis virus, Sacbrood virus and DWV [35–38]. Plasmid standards have also been designed for the analysis of DWV-B IRES activity and DWV-B/DWV-A recombination [39,40]. A DWV plasmid assay is the current standard method for DWV quantification detailed in the COLOSS Beebook [37]. A plasmid standard has also been designed to provide *in vitro* transcription of DWV-specific ssRNA for single strand RT-qPCR analysis, and a linearized RNA transcript [41,42]. Recently, a standard reference plasmid has been designed containing fragments of six *A*. *mellifera* viruses (Acute bee paralysis virus, Black queen cell virus, DWV, Israeli acute paralysis virus, Kashmir bee virus, and Sacbrood bee virus) [43].

Most studies looking at DWV infection do not consider the impact that the different variants have on viral load and virulence. In the present study, a new method of quantifying levels of the DWV strains using SYBR Green qPCR has been developed. A single external plasmid standard combining three target areas was designed for the quantification of DWV-A and DWV-B variants, along with the total DWV strain loads. The new assays were assessed on *Apis melifera* and *V*. *destructor* samples and compared favourably with the standard in the COLOSS Beebook method [34].

## Methods and materials

### DWV variant plasmid quantification primer design

**Fig 1:**
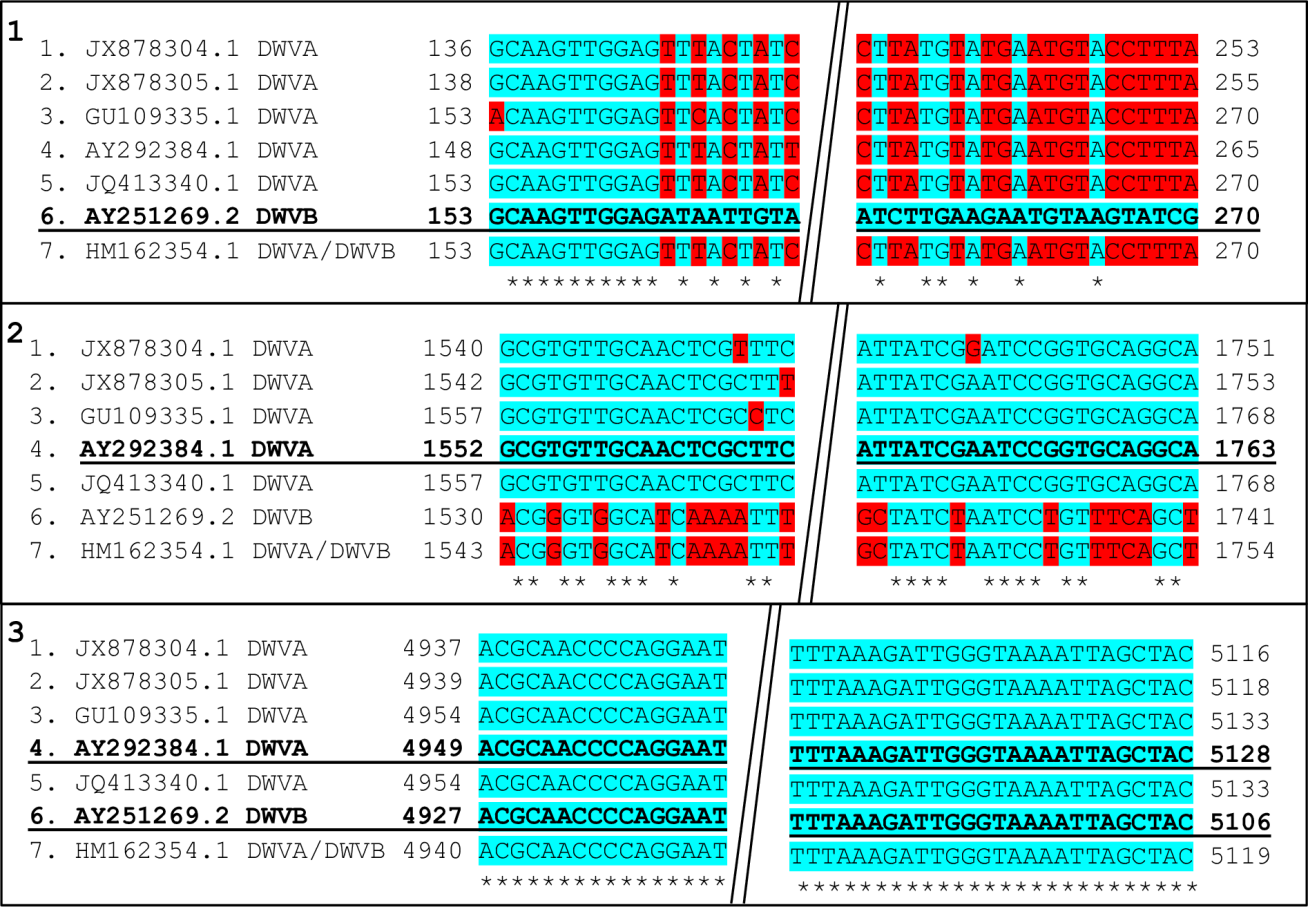
Location of quantification viral primers. 1) DWV-B primer location, 2) DWV-A primer location, 3) Pan-DWV primer location, shown on all seven viral sequences used. Bold and Underlined sequences identify which sequence was used for primer design, numbers correspond to location on genome sequences (Forward primers on the left, Reverse on the right), blue highlighted (*) areas indicate consensus and red areas indicate mismatch locations, diagonal double line break indicates removed sequence section between primer locations.

Three sections within the DWV genome were identified to allow quantification of both the DWV-A and DWV-B variants, and a conserved section for multiple DWV variants (Pan-DWV). All primers designed (S1 and S2 Tables) for this plasmid assembly were designed in Primer3Plus [44]. Seven DWV-A and DWV-B strain sequences from multiple countries (JX878305.1, JX878304.1, GU109335.1, AY292384.1, JQ413340.1, AY251269.2, and HM162354.1) were aligned in Mega 6.06 and Clustal Omega [45–47], to aid in the design of the variant specific and conserved sections for use in plasmid assembly. These sequences from a range of countries were chosen to aid the capture of the most viral diversity. The DWV-A variant specific section is located in a conserved region across DWV-A variants (1427-1895nt, AY292384.1, within the VP2 capsid protein region); a larger (468bp) section was designed for plasmid insertion, containing a smaller section (211bp) for quantification primers (DWV-A) (Fig 1). A large DWV-B variant insert (357bp) (7-364nt, AY251269.2, within the nontranslated region (NTR) containing the internal ribosome entry site (IRES) region) was designed to include an 116bp section used for quantification (DWV-B) (VDV-1 IRES primers taken from [25] (Fig 1). A completely conserved region (Pan-DWV section) across all seven sequences within the helicase protein region was used in primer design to allow maximum variant capture. A large insert (375bp) (4883-5257nt, AY292384.1) was designed to include a 179bp section used for quantification (Pan-DWV) (Fig 1). All three sequences were aligned using Mega version 6.06 [47] and analysed *in silco* to ensure that they were unlikely to cross-hybridise.

### Variant-specific primer design

To test for variant specificity, tagged primers were created for the variant specific DWV-A and DWV-B primer sites, by designing primers that would amplify the opposite variant to the primers intended target (DWV-A variant specific (DWVA_VS) and DWV-B variant specific (DWVB_VS) (S1 Table). These primers were designed due to the close nucleotide identity between DWV-A and DWV-B, to ensure the variant specificity of the assay. The primers were designed to detect the mismatched sequences in the quantification primer sites (Fig 1). The DWV-A variant specific primers were designed to match the sequence 6 (Panel 2, Fig 1) and the DWV-B variant specific primers were designed to match sequence 5 (Panel 1, Fig 1). These variant-specific primers were used to create variant-specific plasmids to allow the assay quantification primers to be tested. RNA was extracted using TRIzol® Reagent (Sigma-Aldrich, Gillingham, UK) from nine individual worker *A*. *mellifera* collected from the University of Aberdeen apiary. cDNA was synthesized using iScript™ cDNA Synthesis Kit (Bio-Rad Laboratories, USA) from 500ng RNA per sample, and then pooled to allow the viral primers to capture as many variants as possible for inserts. PCR was performed for each primer set using 25μl BioMix Red (Bioline Reagents Limited, London, UK), 1μl 10μM primer sets, 22μl H_2_O and 2μl template cDNA (1/5 dilution). The following PCR profile was used: 15 minutes at 95°C, followed by 36 cycles of 30 seconds at 94°C, 1 minute at 58°C, 30 seconds at 72°C, followed by 10 minutes at 72°C. PCR products were analysed by gel electrophoresis on 1% agarose Tris-borate-EDTA (TBE) gel with SYBR® safe DNA gel stain (ThermoFisher Scientific, Loughborough, UK). The resultant PCR products (DWVA_VS and DWVB_VS) were subsequently used in a TOPO® TA Cloning reaction for plasmid insertion using the pCR®4-TOPO plasmid (Invitogen, Life technologies, UK). Plasmids were transfected into *Escherichia coli* competent cells (JM109) (Promega, Southampton, UK), and bacterial colonies grown overnight on LB agar with ampicillin (100mg/ml). Colony picks and PCR was performed to confirm plasmid insert uptake using insert primers (DWVA_VS and DWVB_VS primers, respectively) (S1 Table). Positive colonies were grown overnight in LB broth with ampicillin and the plasmid preps were subsequently isolated using a QIAprep® Spin Miniprep Kit (Qaigen, Manchester, UK). PCR was performed using the variant specific primers to ensure plasmid preps contained the correct insert (S1 Table).

To check the specificity of the assay quantification primers (DWV-A and DWV-B) (S2 Table), qPCR was carried out using the variant-specific plasmids and *A*. *mellifera* cDNA (1/100 dilution) as positive controls for the assay primers. The DWV-A variant-specific plasmid (1/50 dilution) was tested against the DWV-A primers, and the DWV-B variant-specific plasmid (1/50 dilution) was tested against the DWV-B primers. If assay primers were specific as designed then no amplification product would be expected with the variant-specific plasmids as template.

All qPCRs were carried out using the Bio-Rad CFX Touch Real-Time PCR detection system, and all analyses performed with the Bio-Rad FCX Manager (Bio-Rad). SYBR Green fluorescence detection was used and all samples were done in triplicate in 20μl reactions containing 10μl iTaq Universal SYBR Green Supermix (Bio-Rad), 1μl of 10mM primer sets, 4μl H_2_O and 5μl template cDNA, per primer using the following qPCR profile: 3 minutes at 95°C, followed by 36 cycles of 15 seconds 94°C, 30 seconds of 58°C, and 15 seconds of 72°C, followed by 10 minutes at 72°C, and a melt curve from 65°C to 95°C increasing 0.5°C every 5 seconds.

### DWV variant plasmid assembly

Following the successful testing of the primer specificity through the production of variant specific plasmids, three individual plasmids (DWV-A, DWV-B and Pan-DWV) were created using the cloning protocol described above. These three plasmids were created to allow all three primer sets to be used with each plasmid to ensure there was no cross-reactivity prior to DWV variant plasmid construction. Custom T3 and T7 forward and reverse DNA sequencing (Eurofins Genomics, Germany) was carried out on all three plasmids. Sequence identity was confirmed to be either DWV-A (Pan-DWV and DWV-A plasmid) or DWV-B (DWV-B plasmid) following BLASTn searches. Sequences were aligned in MEGA 6.06 to ensure they matched predicted sequences prior to DWV variant plasmid assembly. The three inserts were arranged using the NEBuilder Assembly Tool (New England BioLabs (UK), Hitchin, UK) to enable a Gibson assembly reaction to be used combing all three sections in a pCR®4-TOPO plasmid (S1 Fig). Inserts were generated using Gibson assembly PCR primers (S1 Table) and annealing temperature (60°C) using the PCR profile given above. Following insert generation the Gibson Assembly reaction was carried out following the manufacturer’s instructions for 2–3 fragment assembly (New England BioLabs). This initial product was then used as template in a subsequent PCR reaction to increase the starting material (DWVL_F and DWVBL _R). The amplified PCR product was then cloned into JM109 *E*.*coli* competent cells and plasmid preps were produced as described above. PCR was carried out on plasmid preps using DWVL_F and DWVBL_R primers along with variant specific qPCR primers, with positive samples being confirmed via DNA sequencing (Eurofins Genomics) and qPCR analysis. Positive plasmid prep samples were pooled and concentration quantified using a Nanodrop ND-1000 microspectrophotometer (Thermoscientfific, Loughborough, UK). Exact genome equivalents (ge) were calculated using the following formula (http://www6.appliedbiosystems.com/support/tutorials/pdf/quant_pcr.pdf); based on molecular weight of the plasmid and plasmid DNA concentration, to produce standard stock solutions of known genome equivalent concentration. To test the linearity of the single construct plasmid assay, standard curves for all three primer pairs were created ranging from 100,000,000 to 100 plasmid copies per qPCR reaction. Linearity was also tested using *A*. *mellifera* cDNA to create a serial dilution containing six dilutions of pooled *A*. *mellifera* (1/5 dilution) of cDNA. All linearity test qPCRs were performed in 10ul reactions (5μl iTaq Universal SYBR Green Supermix (Bio-Rad), 0.5μl of 10μl primer sets, 2μl H_2_O, 2.5μl template cDNA). All standards and cDNA samples were assayed in quadruple using the thermocycler profile conditions, described above.

The limit of detection (LOD) for each primer set was calculated via the LOD_95%_ method, using 75, 50, and 25 plasmid copies per qPCR reaction based on the lowest quantity that can be detected in at least 95% of the wells [48]. LOD was tested on 10 samples of each dilution quantity. PCR products from *A*. *mellifera* cDNA from the University of Aberdeen Apiary using the Pan-DWV, DWV-A or DWV-B primers (12 samples per primer pair). PCR products were purified using a QIAquick PCR Purification Kit (Qiagen, Manchester, UK) and were sent for primer specific-sequencing (Eurofins Genomics, Germany), to confirm all three primer pairs were only detecting DWV variants.

### DWV variant plasmid external standard qPCR assessment on biological samples

To assess the plasmid and assays for absolute quantification in biological samples, *A*. *mellifera* worker samples were collected from the University of Aberdeen Apiary (Newburgh, Aberdeenshire, NJ9926), two apiaries in Aberdeenshire (Inverurie, Aberdeenshire), an apiary in the *V*. *destructor*-free Beinn Eighe National Nature Reserve (Anancaun, Ross-shire, NH0262), and four apiaries in South East England (Essex, Bedfordshire, and two locations in Suffolk). *V*. *destructor* samples were collected from brood combs from the University of Aberdeen apiary. All samples from the Aberdeen area were collected and stored at -80°C within hours. All other samples were transported to Aberdeen in RNAlater (Sigma-Aldrich) and then stored at -80°C. Heads were removed from *A*. *mellifera* prior to extraction as bee heads are reported to contain PCR inhibitors [49,50].Bees underwent RNA extraction individually, as described above. *A*. *mellifera* samples were standardised prior to cDNA synthesis to give 500ng RNA per reverse transcription reaction, *V*. *destructor* samples were extracted in pools of three individuals, then standardised prior to cDNA synthesis to give 1000ng RNA per reverse transcription reaction. cDNA was synthesized using iScript™ cDNA Synthesis Kit (Bio-Rad). Sample cDNA (1/5 dilution) was pooled by location for *A*. *mellifera* prior to qPCR analysis. Three pools of three individuals were created for the University Aberdeen, two pools of 4 individuals for Aberdeenshire, and two pools of four individuals for Beinn Eighe. For South East England each location resulted in one pooled sampled, four individuals from Essex, Bedfordshire, and Mid-Suffolk, and two individuals from East-Suffolk. The *V*. *destructor* samples remained separate as three samples of three individuals (1/15 dilution). Comparison of the DWV loads determined by the Pan-DWV assay and the method described in the Beebook [37] based on primers from Genersch, [51] was performed on eight University of Aberdeen samples and eight South East England samples (four from Essex, and four from Mid-Suffolk).

All qPCRs were performed in 20μl reactions (10μl iTaq Universal SYBR Green Supermix (Bio-Rad), 1μl of 10μM primer sets, 4μl H_2_O, 5μl template cDNA (1/5 dilution of cDNA for all *A*. *mellifera* samples, or 1/15 dilution of cDNA for all *V*. *destructor* samples). Standards and unknown samples were assayed in triplicate using the thermocycler profile conditions described above. New dilutions for plasmid standard curves were prepared from stocks for each assay. Viral loads of samples were calculated using the linear regression equation from the standard curve (Log_10_.ge *vs* Cq value) from the DWV variant plasmid. Five individuals from the University of Aberdeen were analysed individually to examine the variation between individual *A*. *mellifera* samples.

### Data analysis

All statistical analyses were performed on log_10_ transformed viral titre data, but are presented graphically and in the text in untransformed format. Regression analyses were performed on the data for the plasmid standard curve and serially diluted *A*. *mellifera* cDNA to assess the linear relationship across the range for Pan-DWV, DWV-A and DWV-B. The mean bias and linearity uncertainty (*U*_LINi_) of the assay were determined for each variant section following the methods of Blanchard *et al*. [35].The relationship between DWV-A and Pan-DWV titres in five *A*. *mellifera* from the University of Aberdeen was determined by calculating the Pearson’s correlation coefficient. Comparison of the DWV titres determined by the Beebook Method [34] and the Pan-DWV method was assessed by a two-tailed 1-sample Z-test of the relative DWV titre [(Pan-DWV / Beebook Method)*100]. Statistical tests were performed in Minitab version 18.

## Results

### Specificity, linearity and sensitivity of the DWV-A, DWV-B, and DWV assays

*In silco* analysis was performed on all three variant qPCR amplicons to ensure all sequence similarities were within DWV variants. The seven DWV sequences presented in Fig 1 and a further seven complete DWV sequences (KX373899.1, KY909333.1, AJ489744.2, KT004425.1, KU847397.1, MF036686.1, KX783225.1) were analysed *in silco* for primer specificity. The Pan-DWV primer sites were found in all 14 sequences, the DWV-A primer sites were found in all DWV-A sequences either as a perfect match or with one base mismatches (none within the 3’ end) with the exception of one sequence which had two mismatches. The DWV-B primer sites were conserved in all the DWV-B sequences.

When the DWV-A-specific primers were tested with the plasmid construct containing the DWV-B variant of that VP2-capsid region, there was no detection of any product (Ct > 35 cycles, no determinable melting point). Likewise, the DWV-B specific primers detected nothing with the plasmid containing the DWV-A variant of that IRES region. Conversely, the DWV-A specific primers readily amplified a product from the plasmid containing the DWV-A variant of the VP2-capsid region and the DWV-B primers amplified a product from the plasmid containing the DWV-B variant of the IRES region. Thus, the DWV-A and DWV-B primers demonstrated specificity for the appropriate DWV strain.

**Fig 2:**
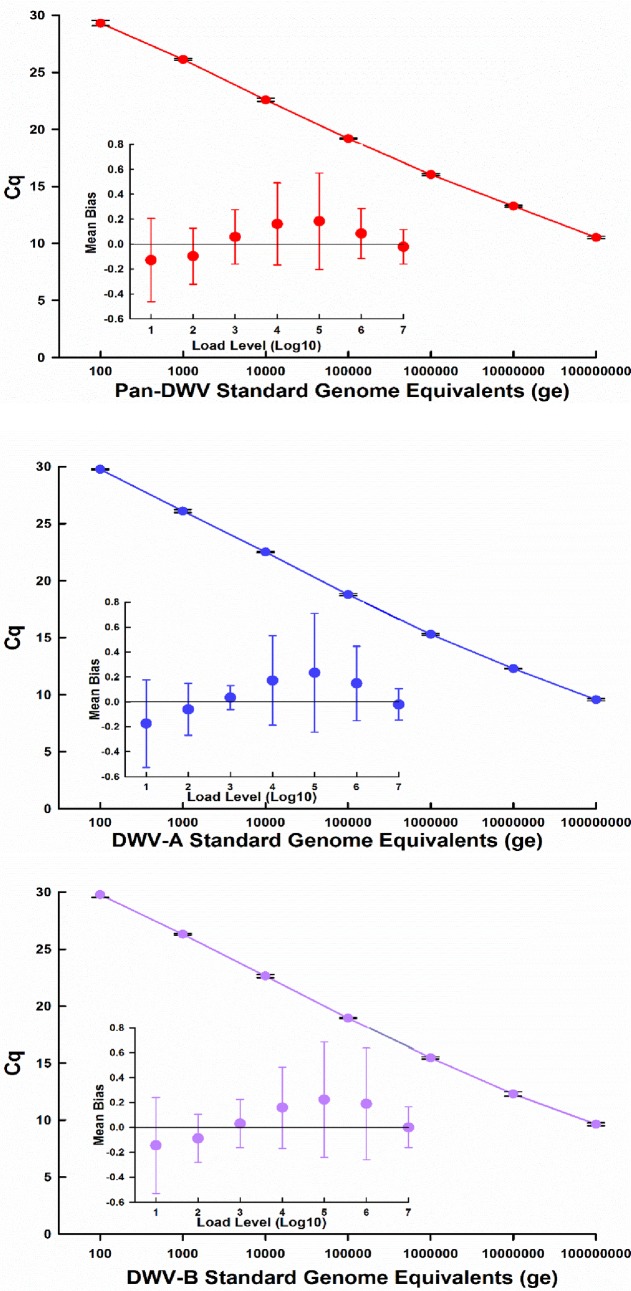
Standard curves and linear uncertainty plots for Pan-DWV, DWV-A and DWV-B assays. Standard curves and linearity uncertainty (*U*_LINi_) plots for each primer pair using the single constructed plasmid external standard containing fragments for A.) Pan-DWV, B.) DWV-A and C.) DWV-B. Data for the standard curve represent mean ± SE performed in quadruple. Linear regression performance was determined by calculating the mean bias for each load, with bars representing the linearity uncertainty (*U*_LINi_).

The plasmid sections were determined to have good efficiencies (95.7%-109%) producing dynamic linear curves across a large range (100–100,000,000 plasmid copies per qPCR reaction) for all three variants DWV-A, DWV-B and Pan-DWV contained within the single constructed plasmid (Pan-DWV R^2^ = 0.997, DWV-A R^2^ = 0.997, and DWV-B R^2^ = 0.996, Fig 2). The mean bias and linearity uncertainty (*U*_LINi_) of the assay were determined for each variant section, as described by Blanchard *et al*. [35]. For all three qPCRs, the absolute mean bias values at all concentrations tested were ≤ 0.25 log_10_.

The limit of detection for each DWV variant was empirically determined [48], indicating the lowest amount of copies per qPCR reaction which can be detected in at least 95% wells. The lowest viral loads that can be reproducibly determined were: Pan-DWV = 50, DWV-A and DWV-B both = 25 plasmid copies per qPCR reaction. Each primer set gave a specific melt curve following qPCR, these temperatures were different for each amplicon (DWV-A = 77 ± 0.042°C; DWV-B = 74.8 ± 0.071°C; Pan-DWV = 80.2 ± 0.74°C), allowing each section to be easily identified during analysis.

The assay’s linearity was also assessed with a serial dilution series (neat to1:100,000 dilution) of 1/5 dilution of cDNA produced from a pool of nine University of Aberdeen *A*. *mellifera*. The serial dilution series displayed linearity (S2 Fig) for all three variant targets over a wide dynamic range (Pan-DWV R^2^ = 0.998, DWV-A R^2^ = 0.998, and DWV-B R^2^ = 0.996). PCR products from *A*. *mellifera* cDNA from the University of Aberdeen Apiary using the Pan-DWV, DWV-A or DWV-B primers (12 samples per primer pair) were sequenced and homologues in GenBank retrieved by BLASTn and aligned. In all three cases, the primer pair used generated an amplicon whose sequence matched the expected target confirming discriminatory specificity for the DWV-A and–B qPCR assays and no false negatives with the Pan-DWV primers.

### DWV variant composition in individual *A*. *mellifera* and *V*. *destructor*

**Fig 3:**
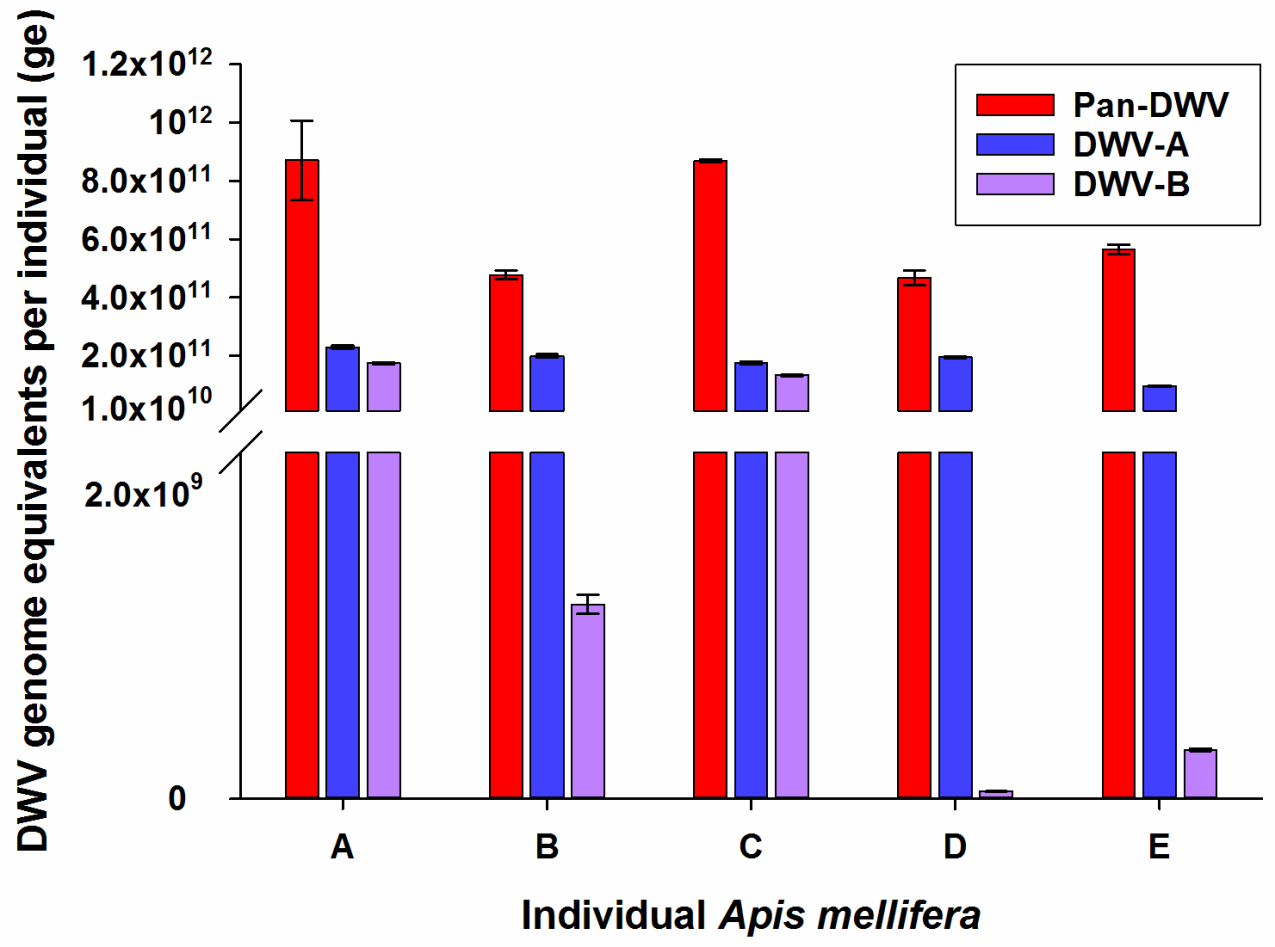
DWV-A, DWV-B and Pan-DWV in *A*. *mellifera* from heavily Varroa-infested hives. The DWV-A, DWV-B and Pan-DWV titres in five individual *A*. *mellifera* from the University of Aberdeen apiary employing the single plasmid construct as the external standard. Data presented are DWV genome equivalents per individual (total extracted RNA) mean ± SE performed in triplicate, n = 5.

Five individual *A*. *mellifera* workers from the University of Aberdeen apiary were analysed to determine the level of variation in the DWV variant composition in individual bees (Fig 3). Even within this single colony there was great variation in the relative proportion that the DWV-A and DWV-B contributed to the total Pan-DWV titre. For example, individuals A and C had almost equal DWV-A and DWV-B loads, while D and E had much higher proportions of DWV-A than DWV-B (Fig 3). The total Pan-DWV titre was always greater than the sum of the variant restricted DWV-A and DWV-B titres with approximately 40% of the total Pan-DWV not accounted for by the sum of DWV-A and DWV-B (Fig 3). Importantly, we determined that total DWV viral load could not be extrapolated from the DWV-A variant specific primer (Pearson’s coefficient correlation r = 0.199, P = 0.749).

**Fig 4:**
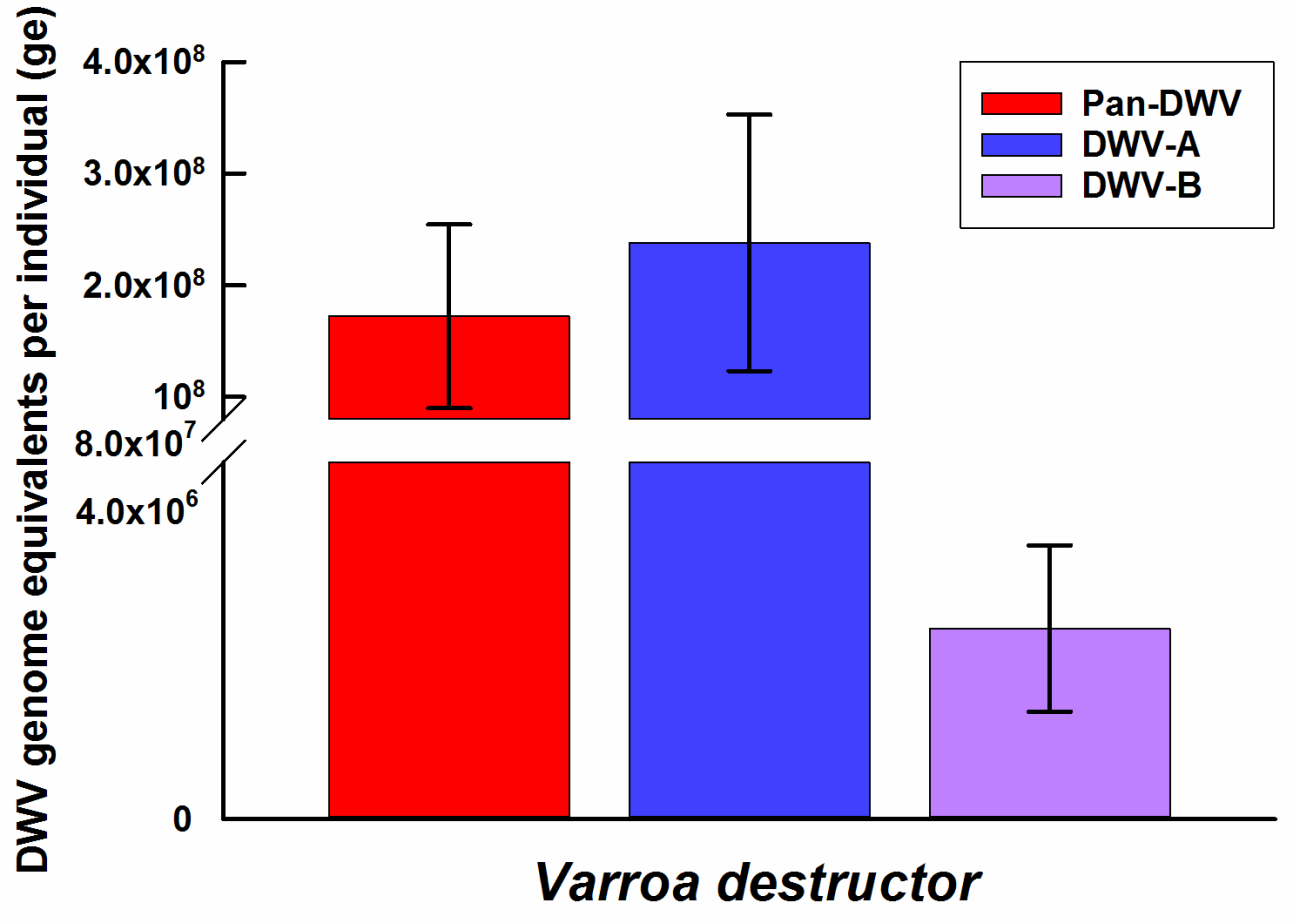
DWV-A, DWV-B and Pan-DWV in *Varroa destructor*. The average DWV-A, DWV-B and Pan-DWV titres in *V*. *destructor* from the University of Aberdeen apiary employing the single plasmid construct as the external standard. Data presented are DWV genome equivalents per mite (total RNA from pooled extractions), mean ± SE performed in triplicate of three pools each containing three mites.

*V*. *destructor* collected from the University of Aberdeen apiary were analysed to assess the applicability of the DWV-A, DWV-B and Pan-DWV primers and constructed plasmid external standard to determine the variant composition of DWV within the mites. All three DWV variants were readily detectable within the *V*. *destructor* and the total viral load determined with the Pan-DWV primers accounted for the sum of the DWV-A and DWV-B loads (Fig 4). DWV-A was far more predominant than DWV-B within these *V*. *destructor* samples.

### Determining DWV composition in *A*. *mellifera* in different regions and *V*. *destructor* infestation levels

**Fig 5:**
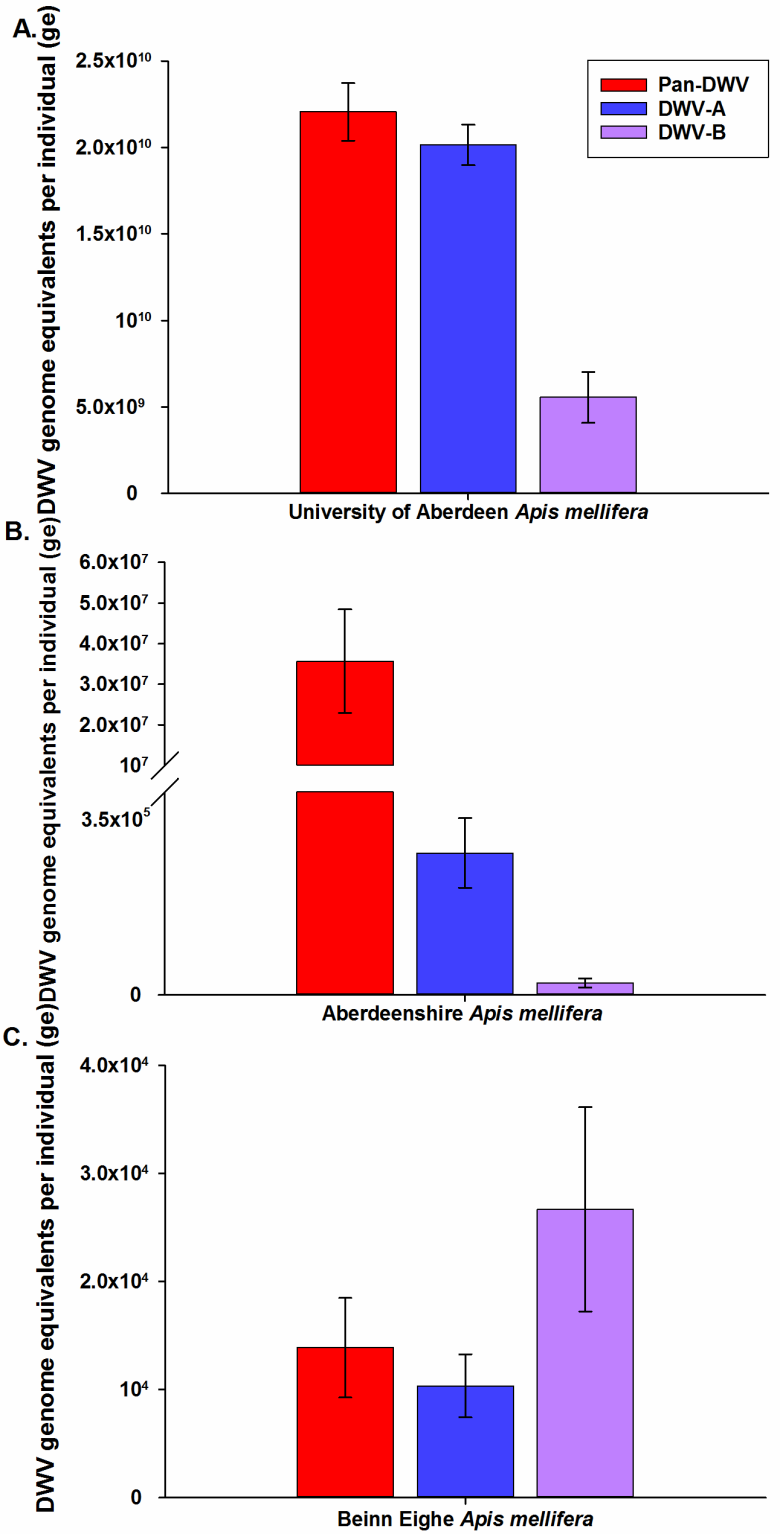
DWV-A, DWV-B and Pan-DWV levels in *A*. *mellifera* from hives with differing Varroa infestations. DWV-A, DWV-B and Pan-DWV titres in *A*. *mellifera* from three apiaries in Scotland, A) Heavily Varroa-infested colonies at the University of Aberdeen apiary (n = 9), B) Lowly Varroa-infested colonies at an Aberdeenshire apiary (n = 8), and C) Varroa-free colonies at the Beinn Eighe National Park (n = 8). Data presented are DWV genome equivalents per individual (total extracted RNA), mean ± SE performed in triplicate.

Viral loads were determined in adult worker *A*. *mellifera* from different regions within the UK and apiaries containing different infestation levels of *V*. *destructor* with all three newly developed assays. In every *A*. *mellifera* analysed, both DWV-A and DWV-B were detected, but in widely varying proportions (Fig 5). The Pan-DWV primers consistently detected the highest level of viral load and, in general, this was equal to or greater than the sum of the DWV-A and DWV-B viral loads. *A*. *mellifera* from the heavily Varroa-infested colonies at the University of Aberdeen apiary which undergo minimal *V*. *destructor* control had extremely high levels of total Pan-DWV, which largely consisted of the DWV-A variant (91%) (Fig 5A), which was also seen in the *V*. *destructor* data from this apiary (Fig 4). The Aberdeenshire *A*. *mellifera* which are of the same origin and stock as the University of Aberdeen samples, but undergo standard *V*. *destructor* treatment and have relatively low *V*. *destructor* infestation levels, contained 500X less Pan-DWV (>40,000,000ge vs <20,000,000,000ge, T_(df = 1)_ = 4.98, P = 0.126) compared to the University of Aberdeen samples (Fig 5B). *A*. *mellifera* from Beinn Eighe, a totally *V*. *destructor*-free region, had extremely low total Pan-DWV consisting of approximately equal levels of DWV-A and DWV-B (Fig 5C). Pan-DWV levels were approximately 1.5 million times lower in the *V*. *destructor*-free *A*. *mellifera* than the University of Aberdeen samples (>14,000ge vs <20,000,000,000ge, T_(df = 1)_ = 29.5, P = 0.022). Interestingly, the *V*. *destructor*-free *A*. *mellifera* had relatively higher proportions of DWV-B than DWV-A than *V*. *destructor* infested *A*. *mellifera* (Fig 5A–5C).

**Fig 6:**
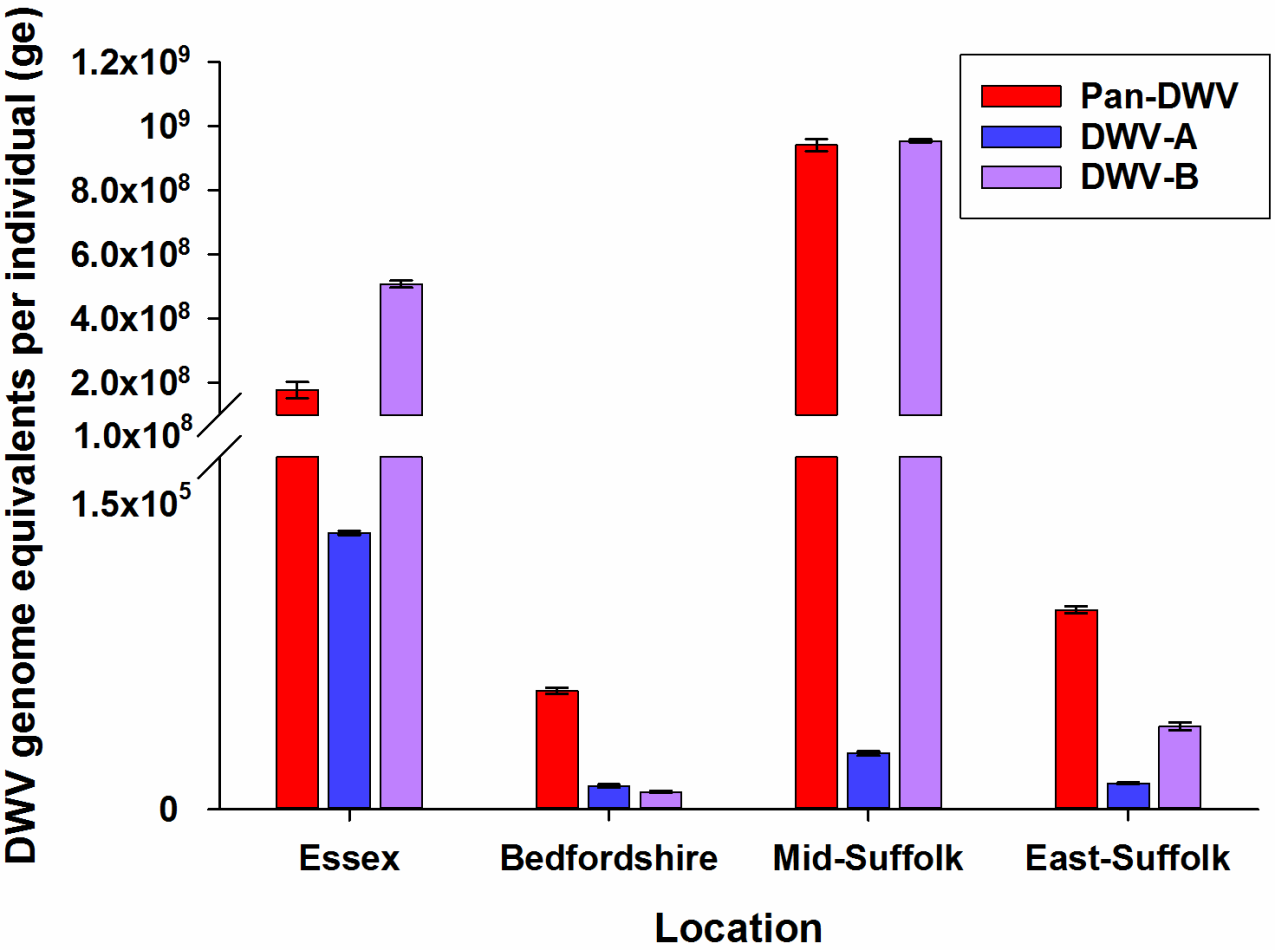
DWV-A, DWV-B and Pan-DWV levels in *A*. *mellifera* in some English colonies. DWV-A, DWV-B and Pan-DWV titres in *A*. *mellifera* from four apiaries in South East England (Essex n = 4, Bedfordshire n = 4, Mid-Suffolk n = 4, East-Suffolk n = 2) employing the single plasmid construct as the external standard. Data presented are DWV genome equivalents per individual (total extracted RNA), mean ± SE performed in triplicate.

Additionally, we determined the total and variant specific DWV loads in *A*. *mellifera* workers in four locations in South Eastern England in apiaries which undergo standard *V*. *destructor* treatments and to maintain low *V*. *destructor* levels. Across the four locations, total Pan-DWV loads varied considerably and all colonies contained both the DWV-A and DWV-B variants (Fig 6), with both Essex and Mid-Suffolk containing proportionally higher DWV-B levels. Notably, it was obvious that determining only the DWV-A variant would have greatly underestimated the total DWV load in all of the South East England *A*. *mellifera* samples (Fig 6).

### Standard Beebook Method vs new Pan-DWV method

**Fig 7:**
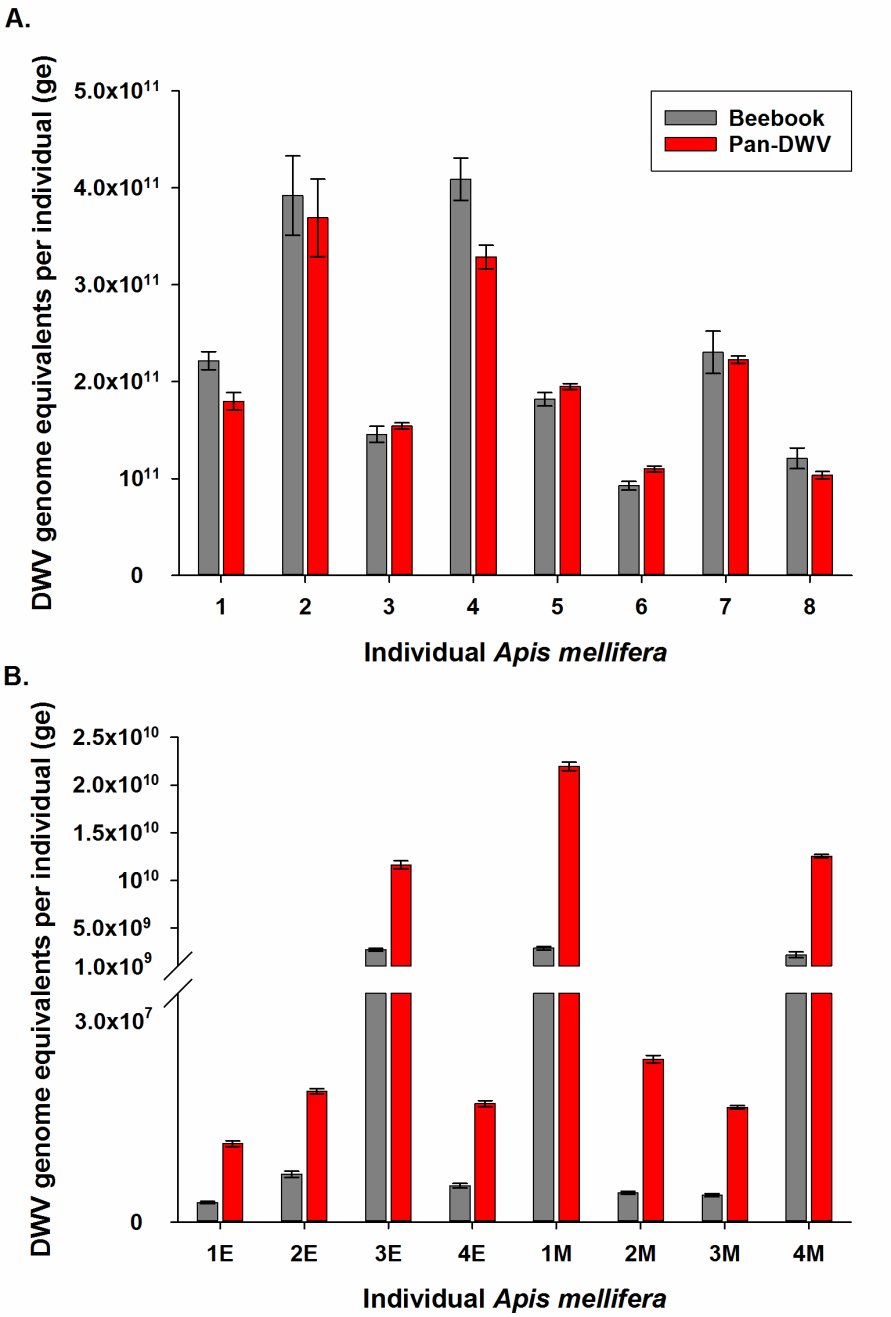
Comparison of the Beebook Method and the Pan-DWV method total DWV titre determination. Total DWV titres in individual *A*. *mellifera* from two locations with different dominant strains as determined by the Beebook Method and the Pan-DWV method. A) University of Aberdeen apiary (DWV-A dominant, n = 8) and B) South East England (E = Essex, n = 4; and M = Mid-Suffolk, n = 4) (DWV-B dominant). Data presented are DWV genome equivalents per individual (total extracted RNA), mean ± SE performed on triplicates.

We compared the Beebook Method to the Pan-DWV primer within our assay by analysing eight individual University of Aberdeen *A*. *mellifera* and eight individuals from South East England (four from Essex, and four from Mid-Suffolk) (Fig 7). These locations were selected to highlight the impact of strain composition (DWV-A dominant at the University of Aberdeen (Fig 3), and DWV-B dominant in the South East England sites (Fig 6)) on the estimated viral titres using the Beebook method and Pan-DWV primers. In all University of Aberdeen samples from a DWV-A dominant hive, similar viral loads were determined using both the Beebook Method and Pan-DWV primer (Fig 7A) (96.2 ± 4.9% (95% CI 86.7–105.7%), Z = -1.41, P = 0.160). However, in the South East England samples from DWV-B dominant hives, the Pan-DWV primer determined consistently and significantly higher viral loads compared to the Beebook Method (Fig 7B) (471.4 ± 55.8% (95% CI 362–581%), Z = 14.60, P < 0.0001).

## Discussion

As for most viruses, DWV’s existence as variant strains proves a challenge for the accurate quantification of viral levels with *A*. *mellifera* and *V*. *destructor*. Within the literature, research into DWV can be broadly split, with some focusing solely on detecting titres of specific variant strains, but the majority of studies are interested in reporting total DWV titres [52–54]. Here we report on the development and field-testing of assays for the analysis and quantification of the total DWV (Pan-DWV) and the variant specific DWV-A and DWV-B titres. All assays utilized the same plasmid as the external standard for absolute quantification which contained three DWV viral sequences that can be used to discriminate between DWV-A and DWV-B populations, while also suitable for detecting the majority of the total DWV population (Pan-DWV). The proposed assays have low limits of detection; DWV-A and DWV-B (LOD_95%_≥25 plasmid copies per qPCR reaction), and Pan-DWV (LOD_95%_≥50 plasmid copies per qPCR reaction), a wide dynamic range (100–100,000,000 plasmid copies per qPCR reaction) with each of the three amplicons having distinct melting points allowing easy identification of the product. Despite the high nucleotide identity (~84%) that occurs within DWV [16]; each variant-specific section selected and the corresponding primer pairs designed did not cross-react with the other variant. The ability to join multiple viral strains in one combined plasmid has been previously shown [43], with a plasmid combining six different viral sections together, indicating the suitability of this method especially for viral strain investigations. The assay gave similar total viral titres at the individual level, to previously published data on DWV viral titres in *A*. *mellifera* with varying levels of *V*. *destructor* infestation [23,28].

All three assays rely on a single plasmid construct, resulting in only one standard source having to be prepared, purified, stored and diluted, while allowing it to remain equimolar across assays. Further plasmid or transfected *E*. *coli* cells can be readily generated in any lab from the easily shipped original plasmid and shared between researchers. If desired, it should be possible to design a probe-based, multiplexed assay to allow DWV-A, DWV-B and the total DWV titre to be determined in a single qPCR reaction using the regions of conservation and the external plasmid identified in this report. Its demonstrated success in samples from across the whole UK and even in *A*. *mellifera jemenitica* in Saudi Arabia (A. Hroobi, unpublished observations) suggest it will be applicable over a very wide geographic range, if not world-wide.

The currently described method for DWV titre determination [37] is presented in the COLOSS Beebook chapter “Standard methods for molecular research in *Apis mellifera*”. The Beebook Method utilizes primers that were designed in 2005 [51] and have been employed in several studies [20,55]. However, much research in the area of DWV has taken place since 2005 and our understanding of the DWV variants has increased [15,16,23,25,26,28]. It is now accepted that there are two clear DWV variants, the classic DWV (DWV-A) and the VDV-1 strain (DWV-B), and recombinants between these two main variants [16,23,25,26]. DWV-B has been reported to be far more virulent to *A*. *mellifera* than DWV-A [26] and it is clearly important in modern DWV studies to be able to consider the tires of DWV-A, DWV-B and the total DWV. The assays presented in this paper have been demonstrated to be able to deliver this requirement of separating the two main variants. While the assays are not designed for recombinant quantification, the Pan-DWV section will detect any recombinants which possess that section of the genome.

The performances of the new assay were compared to the Beebook Method on *A*. *mellifera* samples from two geographic locations with different dominant strains, the University of Aberdeen apiary (DWV-A dominant) and two locations in South East England (DWV-B dominant). For the Beebook Method, a plasmid standard was created and primers used as described in the Beebook [37]. In DWV-A dominant hives similar viral titres were obtained with both methods, while in DWV-B dominant hives the Pan-DWV method gave significantly higher viral titres. The differences in viral titres from the DWV-B dominant hives show that the Pan-DWV primers are designed in a highly conserved region of both the DWV-A and DWV-B genomes, while the Beebook primers are located in a region that is conserved within the DWV-A genome but not the DWV-B genome. These differences in conserved regions explain why the Pan-DWV primer is more efficient at detecting DWV-B, when compared to the Beebook Method. The Beebook Method primers were designed in 2005, when we had a less complete picture of the DWV strain diversity, resulting in an understandable DWV-A bias. However, following more recent discoveries into the relationship between DWV-A and DWV-B, methods for total viral titres need to take both strains into account. These results indicate that while the Beebook Method works well in DWV-A dominant samples, in DWV-B dominant samples this method will underestimate total viral titres. Sequences of the amplicons generated by the Pan-DWV primers confirmed that this method was only detecting DWV variants.

The assays described in this report provides a more in-depth analysis of viral infection allowing DWV variant-specific information and total DWV titres to be collected by a qPCR approach. Though the qPCR approach does not provide anywhere near the information that deeper sequencing can provide [25–28], it does allow far more samples to be analysed at a fraction of the cost or time and is, thus, suited to bigger surveys. The DWV-B variant is proposed to be the more pathogenic DWV strain [26], therefore it is important to be able to determine the DWV-A: DWV-B proportions and to investigate DWV-B’s virulence in multi-apiary and multi-colony wide surveys in the field. The titres of DWV-A, DWV-B, and the Pan-DWV were determined in individual *A*. *mellifera* at the University of Aberdeen apiary and showed a wide variation between the levels of DWV-A and DWV-B. The DWV variant profile was similarly determined in *V*. *destructor* at this apiary, with similar dominant variant distribution (DWV-A dominant variant) and high viral titres to the *A*. *mellifera* at this site. These similar relationships in DWV structure between *A*. *mellifera* and associated *V*. *destructor* has been previously shown, while the *V*. *destructor* used in this study did not come from the same hive as the *A*. *mellifera* samples, they were from the same apiary [56]. Though not the focus of the present study, it would be interesting to use these DWV assays to investigate possible causes of the varying DWV-A and DWV-B titres in individuals within the same colony relative to age, *V*. *destructor*-status or other variables and the subsequent effect on fitness and mortality. The variation in DWV loads in individual *A*. *mellifera* within the same colony (as seen in this study) have been shown to be related to caste, age and visible condition [57], but these new variant-specific assays would allow deeper investigations.

The multiple UK locations were selected to allow the assays to be tested on samples with different levels of *V*. *destructor* infestation. The University of Aberdeen apiary is minimally treated for *V*. *destructor* and has very high infestation levels, whereas the two sites in Aberdeenshire and the four locations in South East England are regularly treated and have relatively low levels of *Varroa* infestation. Previous research into *V*. *destructor* infestation levels on DWV titres has highlighted the complex relationship between *V*. *destructor* and DWV. DWV titres were much lower in all the sites with relatively low Varroa infestation levels which regularly undergo *V*. *destructor* treatment when compared to the University of Aberdeen apiary which has very high levels of *Varroa* infestation. Treated hives have lower viral loads when compared to untreated hives [58,59] though *V*. *destructor* treatment does not cause an immediate reduction in DWV titres [60]. Additionally, the assay’s suitability for analysis of low viral titre samples was confirmed by analysing bees from a *V*. *destructor*-free location within the Beinn Eighe National Nature Reserve, in the Highlands of Scotland [61]. As expected the DWV viral titre of the Beinn Eighe *A*. *mellifera* was much lower than that found at other sites.

## Conclusion

In conclusion, the three new assays for DWV variant strain quantification described in this report allow for more in-depth analysis of the proposed lesser and more virulent variants (DWV-A and DWV-B, respectively) viral titres in both *A*. *mellifera* and *V*. *destructor* all using the same plasmid construct for the external standard. We have shown that the current standard method, the Beebook method for DWV quantification, underestimates total viral loads when DWV-B strains are substantial within the hives, when compared to the Pan-DWV method. The method for DWV variant strain viral quantification allows the complicated nature of DWV infections to be teased apart at much lower costs and, thus, permitting a much higher number of samples to be processed than more complex deep sequencing approaches.

## Supporting information

S1 Table Oligonucleotide primers used in this study to create plasmids, including sequence, amplicon size, annealing temperature, application in this paper, and reference information. (DOCX)

S2 Table Oligonucleotide primers used in this study for quantification, including sequence, amplicon size, annealing temperature, application in this paper, and reference information. (DOCX)

S1 Fig Schematic illustration of the DWV variant plasmid showing inserted viral sections and primer locations. Forward primers are indicated by black arrows and reverse primers with orange arrows. Viral sections and length are shown above the corresponding sections, and primer product length is given within each section. The grey section shows the location of the T7 binding site, and the green section shows the location of the T3 binding site, to indicate where the inserted sections are location in relation to other features of the pCR4 plasmid.
(DOCX)

S2 Fig Serial diluted *A*. *mellifera* cDNA linearity plot. Linearity plot created using serial diluted pooled *A*. *mellifera* cDNA from the University of Aberdeen apiary, using the single plasmid construct as the external standard. Data presented are DWV genome equivalents per qPCR reaction, mean ± SE performed in quadruple.
(DOCX)
