# Supplementary material for: A real-time PCR method for quantification of the total and major variant strains of the deformed wing virus
Source: PLoS One. 2017 Dec 19;12(12):e0190017. doi: 10.1371/journal.pone.0190017 (PMC5736226; doi:10.1371/journal.pone.0190017)
Supplement: S1 Table — (DOCX) [file pone.0190017.s001.docx]

S1 Table: Oligonucleotide primers used in this study to create plasmids, including sequence, amplicon size, annealing temperature and application in this paper.

| Primer Name | Primer Sequence (5’-3’) | Amplicon size (bp) | Annealing temperature (^o^C) | Application | Reference |
| --- | --- | --- | --- | --- | --- |
| DWV-A_L_F | GTACTCTCCTATATCAGTTTCG | 409 | 58 | Large DWV-A section | Present study |
| DWV-A_L_R | TTTTACGCTCACCGGCGCT |  |  |  |  |
| DWV-B_L_F | CGAATTACGGTGCAACTAAC | 357 | 58 | Large DWV-B section | Present study |
| DWV-B_L_R | GTCACATGGCATTCTACTCT |  |  |  |  |
| Pan-DWV_L_F | TGGACGCGAAATGATGCATTC | 375 | 58 | Large Pan-DWV section | Present study |
| Pan-DWV _L_R | TTCAACAAACGCGCTGCAG |  |  |  |  |
| pCR4_DWVL_F | GCCCTTAAGGGCGAATTCGCTGGACGCGAAATGATGCATT | 405 | 60 | Gibson Assembly Pan-DWV section | Present study |
| DWVAL_DWVL_R | AGGAGAGTACTTCAACAAACGCGCTGCA |  |  |  |  |
| DWVL_DWVAL_F | GTTTGTTGAAGTACTCTCCTATATCAGTTTCGAATAGGTTTG | 429 | 60 | Gibson Assembly DWV-A section | Present study |
| DWVBL_DWVAL_R | CCGTAATTCGTTTTACGCTCACCGGCGC |  |  |  |  |
| DWVAL_DWVBL_F | GAGCGTAAAACGAATTACGGTGCAACTAAC | 387 | 60 | Gibson Assembly DWV-B section | Present study |
| pCR4_DWVBL_R | TAGGGCGAAATTGAATTTAGCTCACATGGCATTCTACTC |  |  |  |  |
| DWVBtagDWVA_F | GCAAGTTGGAGTTTACTATTTTGGATTATG | 129 | 58 | DWV-B tagged with DWV-A | Present study |
| DWVBtagDWVA_R | TAACATTCATACTAAAGGTACATTCATACATAAG |  |  |  |  |
| DWVAtagDWVB_F | ACGGGTGGCATCAAAATTTGTGCGCGAGAAAGTTGTTAGG | 229 | 58 | DWV-A tagged with DWV-B | Present study |
| DWVAtagDWVB_R | ATTGTCCATCTCAGGTTTAGCTGAAACAGGATTAGATAGC |  |  |  |  |
