# Supplementary material for: A real-time PCR method for quantification of the total and major variant strains of the deformed wing virus
Source: PLoS One. 2017 Dec 19;12(12):e0190017. doi: 10.1371/journal.pone.0190017 (PMC5736226; doi:10.1371/journal.pone.0190017)
Supplement: S2 Table — (DOCX) [file pone.0190017.s002.docx]

S2 Table: Oligonucleotide primers used in this study for quantification, including sequence, amplicon size, annealing temperature, application in this paper, and reference information.

| Primer Name | Primer Sequence (5’-3’) | Amplicon size (bp) | Annealing temperature (^o^C) | Application | Reference |
| --- | --- | --- | --- | --- | --- |
| DWV-A_F | GCGTGTTGCAACTCGCTTC | 211 | 58 | DWV-A quantification section | Present study |
| DWV-A_R | TGCCTGCACCGGATTCGATAAT |  |  |  |  |
| DWV-B_F | GCAAGTTGGAGATAATTGTA | 116 | 58 | DWV-B quantification section | (Moore, Jironkin et al. 2011) |
| DWV-B_R | CGATACTTACATTCTTCAAGAT |  |  |  |  |
| Pan-DWV_F | ACGCAACCCCAGGAAT | 179 | 58 | Pan- DWV quantification section | Present study |
| Pan-DWV_R | GTAGCTAATTTTACCCAATCTTTAAA |  |  |  |  |
| Beebook_F | CCTGCTAATCAACAAGGACCTGG | 355 | 58 | Beebook qPCR | (Genersch 2005) |
| Beebook_R | CAGAACCAATGTCTAACGCTAACCC |  |  |  |  |

**References**

GENERSCH, E., 2005. Development of a rapid and sensitive RT-PCR method for the detection of deformed wing virus, a pathogen of the honeybee (Apis mellifera). *Veterinary Journal,* **169**(1), pp. 121-123.

MOORE, J., JIRONKIN, A., CHANDLER, D., BURROUGHS, N., EVANS, D.J. and RYABOV, E.V., 2011. Recombinants between Deformed wing virus and Varroa destructor virus-1 may prevail in Varroa destructor-infested honeybee colonies. *Journal of General Virology,* **92**(1), pp. 156-161.
