# Supplementary material for: A real-time PCR method for quantification of the total and major variant strains of the deformed wing virus
Source: PLoS One. 2017 Dec 19;12(12):e0190017. doi: 10.1371/journal.pone.0190017 (PMC5736226; doi:10.1371/journal.pone.0190017)
Supplement: S1 Fig — Forward primers are indicated by black arrows and reverse primers with orange arrows. Viral sections and length are shown above the corresponding sections, and primer product length is given within each section. The grey section shows the location of the T7 binding site, and the green section shows the location of the T3 binding site, to indicate where the inserted sections are location in relation to other features of the pCR4 plasmid. (DOCX) [file pone.0190017.s003.docx]

S1 Fig


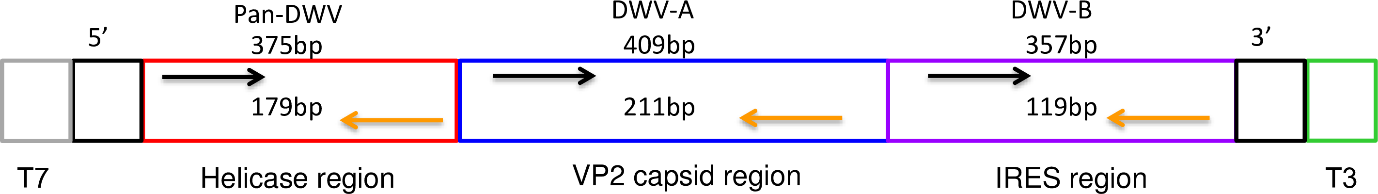


**S1 Fig: Schematic illustration of the DWV variant plasmid showing inserted viral sections and primer locations.**

Forward primers are indicated by black arrows and reverse primers with orange arrows. Viral sections and length are shown above the corresponding sections, and primer product length is given within each section. The grey section shows the location of the T7 binding site, and the green section shows the location of the T3 binding site, to indicate where the inserted sections are location in relation to other features of the pCR4 plasmid.
