# Supplementary material for: A real-time PCR method for quantification of the total and major variant strains of the deformed wing virus
Source: PLoS One. 2017 Dec 19;12(12):e0190017. doi: 10.1371/journal.pone.0190017 (PMC5736226; doi:10.1371/journal.pone.0190017)
Supplement: S2 Fig — Linearity plot created using serial diluted pooled A. mellifera cDNA from the University of Aberdeen apiary, using the single plasmid construct as the external standard. Data presented are DWV genome equivalents per qPCR reaction, mean ± SE performed in quadruple. (DOCX) [file pone.0190017.s004.docx]

S2 Fig

**S2 Fig:** **Serial diluted *A. mellifera* cDNA linearity plot.**

Linearity plot created using serial diluted pooled *A. mellifera* cDNA from the University of Aberdeen apiary, using the single plasmid construct as the external standard. Data presented are DWV genome equivalents per qPCR reaction, mean ± SE performed in quadruple.
